# The effect of increasing heel height on lower limb symmetry during the back squat in trained and novice lifters

**DOI:** 10.1186/s13102-020-00191-y

**Published:** 2020-07-25

**Authors:** Mark G. L. Sayers, S. H. Hosseini Nasab, Caroline Bachem, William R. Taylor, Renate List, Silvio Lorenzetti

**Affiliations:** 1 School of Health and Sport Sciences, University of the Sunshine Coast, Queensland, Australia; 2 Institute for Biomechanics, ETH Zürich, Zürich, Switzerland; 3 Human Performance Lab, Schulthess Clinic, Zürich, Switzerland; 4 Swiss Federal Institute of Sport, Magglingen, Switzerland

**Keywords:** High bar back squat, Heel lift, Symmetry analyses, Statistical parametric mapping

## Abstract

**Background:**

Symmetry during lifting is considered critical for allowing balanced power production and avoidance of injury. This investigation assessed the influence of elevating the heels on bilateral lower limb symmetry during loaded (50% of body weight) high-bar back squats.

**Methods:**

Ten novice (mass 67.6 ± 12.4 kg, height 1.73 ± 0.10 m) and ten regular weight trainers (mass 66.0 ± 10.7 kg, height 1.71 ± 0.09 m) were assessed while standing on both the flat level floor and on an inclined board. Data collection used infra-red motion capture procedures and two force platforms to record bilateral vertical ground reaction force (GRF_vert_) and ankle, knee and hip joint kinematic and kinetic data. Paired t-tests and statistical parametric mapping (SPM1D) procedures were used to assess differences in discrete and continuous bilateral symmetry data across conditions.

**Results:**

Although discrete joint kinematic and joint moment symmetry data were largely unaffected by raising the heels, the regular weight trainers presented greater bilateral asymmetry in these data than the novices. The one significant finding in these discrete data showed that raising the heels significantly reduced maximum knee extension moment asymmetry (*P* = 0.02), but in the novice group only. Time-series analyses indicated significant bilateral asymmetries in both GRF_vert_ and knee extension moments mid-way though the eccentric phase for the novice group, with the latter unaffected by heel lift condition. There were no significant bilateral asymmetries in time series data within the regular weight training group.

**Conclusions:**

This investigation highlights that although a degree of bilateral lower limb asymmetry is common in individuals performing back squats, the degree of this symmetry is largely unaffected by raising the heels. Differences in results for discrete and time-series symmetry analyses also highlight a key issue associated with relying solely on discrete data techniques to assess bilateral symmetry during tasks such as the back squat.

## Background

The relative importance of bilateral limb symmetry to athletic performance has been the focus of several studies in the recent exercise science literature [1–10]. Although a certain degree of symmetry is known to allow optimal performance during cyclical and bilateral tasks [5] bilateral strength symmetries are quite rare, with researchers reporting bilateral asymmetries up to 20% during lower limb tasks in otherwise healthy individuals [11–13]. Accordingly, the numerous studies reporting links between excessive bilateral lower limb strength asymmetries and injury risk address this ‘natural variation’ by using 15% asymmetry as a threshold [11, 12, 14]. Some practitioners question the 15% threshold and adopt a more conservative view, suggesting a 10% threshold is more appropriate, particularly when associated with return to play following injury [15, 16].

Typically, these symmetry thresholds are based on indexes, with the Bilateral Symmetry Index [17] and Symmetry Index (SI) [18] suggested as the only methods to accurately quantify limb strength symmetry during bilateral tasks such as squats, mid-thigh pulls or deadlifts [19]. Similarly, numerous other indexes are used to quantify movement symmetry of peak joint displacement and moment data [for a recent review see 1], with a common approach quantifying symmetry as the difference between the left and right sides (or affected and unaffected limbs, or dominant and non-dominant sides) before expressing these differences as percentages of the average of the two sides [20]. Unfortunately, indexes such as these are based on gross measures of *strength* and/or rely on discrete data, an approach that limits our understanding of the complex interactions occurring between limbs during dynamic tasks [4]. This key limitation can largely be addressed by quantifying bilateral symmetry using time-series based analytical procedures such as Statistical Parametric Mapping (SPM) [21], a procedure that is increasing in popularity in recent biomechanical literature [22–24]. However, at the time of submission of this manuscript the use of SPM to quantify bilateral symmetry during back squatting has not been reported in the scientific literature.

The back squat is a key element in many athletic strength and conditioning programs [25–28], and is considered a symmetrical bilateral movement [29]. This has resulted in the back squat (and variations such as single leg squat and overhead squat) being used frequently as clinical tests of lower limb symmetry [18, 30–36]. For example, clinical research reports asymmetries in lower limb kinetics and/or kinematics during squat exercises in patients following ACL reconstruction [37, 38] and in individuals with long chronic anterior knee pain [7]. Similarly, long jumpers who typically jump using the same limb when performing their sport, also display bilateral differences in peak hip and ankle extension torques when performing back squats [29]. The key assumption inherent throughout much of this research centres around the notion that in healthy populations the lower limbs work symmetrically during squatting style movements. However, this assumption might be flawed, as previous research reports significant bilateral differences of 10–20% in lower limb joint kinetic and kinematic data in asymptomatic individuals during both loaded and unloaded squats [7, 35, 36, 39].

A possible explanation for these asymmetry data may be related to asymmetries in joint range of motion (ROM) and not in force production per sae. For example, the ankle joint is extremely complex [40] and is prone to numerous conditions that limit ankle mobility [41]. Common ankle flexibility and impingement issues limit joint ROM and alter hip and knee kinematics and kinetics during squat exercises [31, 42, 43], with poor ankle mobility is seen as a common cause of incorrect squatting technique [44]. One common approach to address ankle mobility issues is to squat using wedges or weightlifting shoes to elevate the heels (*EH*), with several studies reporting that these modifications alter lower limb kinematics [27, 44–47]. Conversely, the influence of increasing heel height on lower limb squat kinetics is less clear with some researchers reporting no effect for either *EH* or weightlifting shoes [48], while others show clear changes [45, 49]. Differences between these studies may be related to differences in participant cohorts, with the participants in the study by SP Lee, C Gillis, JJ Ibarra, D Oldroyd and R Zane [48] being less experienced than those in the other two projects.

Accordingly, the purpose of this research was to quantify bilateral symmetry in lower limb kinematics and kinetic flexion/extension and ground reaction force data during high bar squatting in regular and novice weight trainers. An additional aim was to examine the influence of various heel lift conditions on sagittal lower limb symmetry in these cohorts. Symmetry analyses were based on a combination of both discrete and time-series analytical techniques. We hypothesize that bilateral lower limb asymmetries will be more common in the novice weight trainers than in the more experienced group. In addition, we hypothesize that artificially raising the heels will reduce asymmetry in both groups.

## Methods

### Participants

Ten males and ten females provided their written informed consent before participation in this project. Participants were divided into two groups (5 females and 5 males per group) based on their weight training experience. The novice group were (age 26.1 ± 4.9 years, mass 67.6 ± 12.4 kg, height 1.729 ± 0.096 m) were familiar with the high-bar back squat exercise but had only just started weight training. The regular weight training group (age 27.6 ± 3.6 years, mass 66.0 ± 10.7 kg, height 1.707 ± 0.090 m) had been weight training 2–3 times per week for several years and had a high-bar back squat single repetition maximum (RM) of at least 1.2x body weight (mean = 1.56 ± 0.32 x body weight). Participants in this group reported that they typically avoided heavy load lifting (loads ≥3RM), focussing most of their squat training around 8–12 RM loads. At the time of data acquisition all participants were free of injury and reported no previous lower limb injuries. This project was approved by the institutional human research ethics committee.

### Data acquisition

**Fig. 1:**
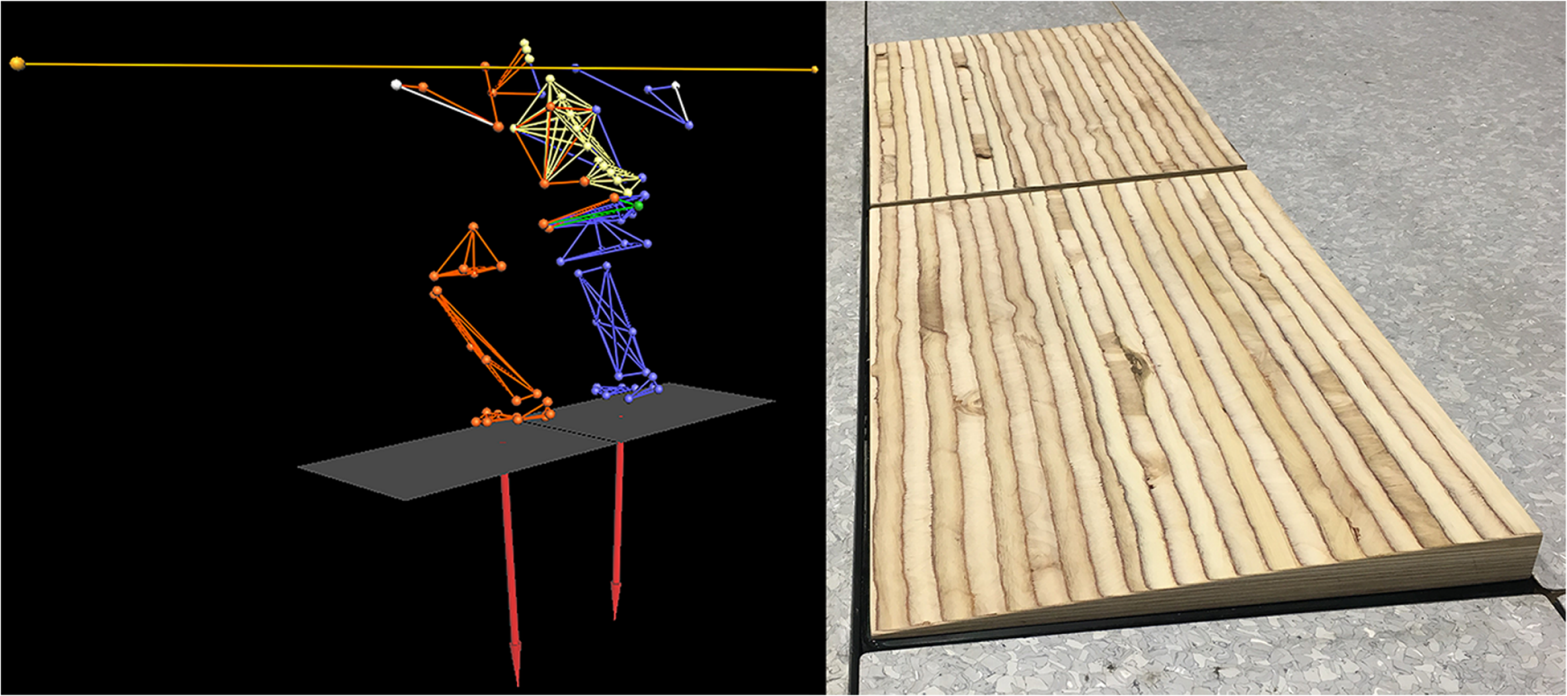
Images showing the machined wedges attached to the two force platforms (left) and the model developed using the Institute for Biomechanics (IfB) marker set (right)

Prior to testing, 77 retro-reflecting markers were attached to the skin adjacent to key anatomical landmarks on the lower limbs, pelvis, trunk and arms [25, 50]. Two additional markers were attached to the end of the barbell (Fig. 1). A static capture was then completed for each participant, with these data used to help define limb lengths during the modelling procedures. Participants then completed a 5 min warm up that included general locomotor activities and several series of 8–12 unloaded squats. Next, participants performed a series of standardised basic motion tasks that were used for defining the functional joint centres of the ankles, knees and hips [50]. Data collection for the flat floor (*FL*) condition involved participants completing sets of eight repetitions of moderate load (50% BW) high-bar back squats while wearing conventional training shoes, standing with each foot on a separate force platform (Kistler Instrument AG, Winterthur, Switzerland). The *EH* condition was created by having the participants stand on two custom-made wooden ramps that were secured to the force platforms (machined to a 4.5° downhill slope – Fig. 1). Each participant was given a standardised set of instructions [25] that encouraged participants to squat down as “low as possible”, with the minimal acceptable depth requiring the upper leg to be at least parallel with the ground. A 2 min rest was provided between sets to minimize possible effects of fatigue [51].

All marker trajectories were tracked throughout data acquisition in three-dimensions (100 Hz) using a 22 camera infra-red motion capture system (MX40 and MX160, Vicon Motion System, Oxford Metrics Ltd., United Kingdom), while the force platforms were sampling at 2000 Hz. The order of the floor conditions was randomised between participants.

### Data analysis

Following capture, rigid body models of the pelvis and lower limbs were reconstructed using the Vicon Nexus software (version 2.4, Oxford Metrics Group, UK). These data were then processed using customised routines in MATLAB (versions 2012a and 2014a, The MathWorks Inc., Natick, MA, USA), with functional joint centres for the lower limbs determined using well established techniques [50]. Standard joint angle coordinate system conventions were used to describe kinematic motions [52], with all positive values and rotations representing flexion. The ankle angle of the *EH* condition was adjusted by the decline angle of the ramp to ensure a consistent reference system across all conditions. It should be mentioned that no filtration was performed on the marker trajectories and ground reaction force data.

A quasi-static inverse dynamics approach was used to determine the three-dimensional external joint moments in the knee, and hip, which were subsequently normalized to the subject’s body mass. This method incorporates ground reaction force data (normalised to body mass), joint centres determined using kinematic data, as well as sex-specific segmental masses [53]. Inertial forces were neglected due to slow accelerations of the segments. The focus was placed on the sagittal plane, as this is the primary plane of motion during the squat movement [25, 28, 50]. A > 0.04 m/s threshold in the vertical velocities of the acromion markers was used to define the start and end points of each single squat cycle [25, 50].

The SI [18] was used as a discrete measure of the symmetry for the vertical ground reaction force data (GRF_vert_). This index quantifies the differences between the maximum values across limbs, expressed as a function of the total value:

$$ SI=\frac{\left( higher\ value- lower\ value\right)}{total\ value}\times 100 $$

The symmetry of peak joint displacement and moment data were calculated as the difference between the left and right sides expressed as a percentage of the average of the left and right sides [20].

### Statistical analyses

Statistical analyses of discrete data were performed using IBM SPSS software (version 24, SPSS AG, Zürich, Switzerland). Multiple repeated-measure analysis of variance (ANOVA) tests were used to analyse the influence of elevating the heels on bilateral limb symmetry. The Greenhouse-Geisser correction was used in cases where data violated the assumption of sphericity. Normality was tested using the Shapiro-Wilk test on the standardised residuals, with the normality assumption fulfilled unless otherwise stated. All significant interactions were followed up with post hoc tests, with Bonferroni corrections performed for multiple comparisons. The relative magnitude of differences in these data were quantified using *Hedge’s g* (*g*) effect size analyses with correction for the small sample sizes used in this investigation. The following descriptors were used to define the magnitude of the effect statistic: < 0.2 = *trivial*, 0.2–0.6 = *small*, 0.6–1.2 = *moderate*, and 1.2–2.0 = *large* [54]. To quantify symmetry in the ankle, knee and hip time-series data, between-limb SPM analyses were completed on angular displacement, joint moment and GRF_vert_ data using the SPM1D technique [55]. Data throughout all analyses are presented as mean ± standard deviation with the significance level set to *P* < 0.05.

## Results

**Fig. 2:**
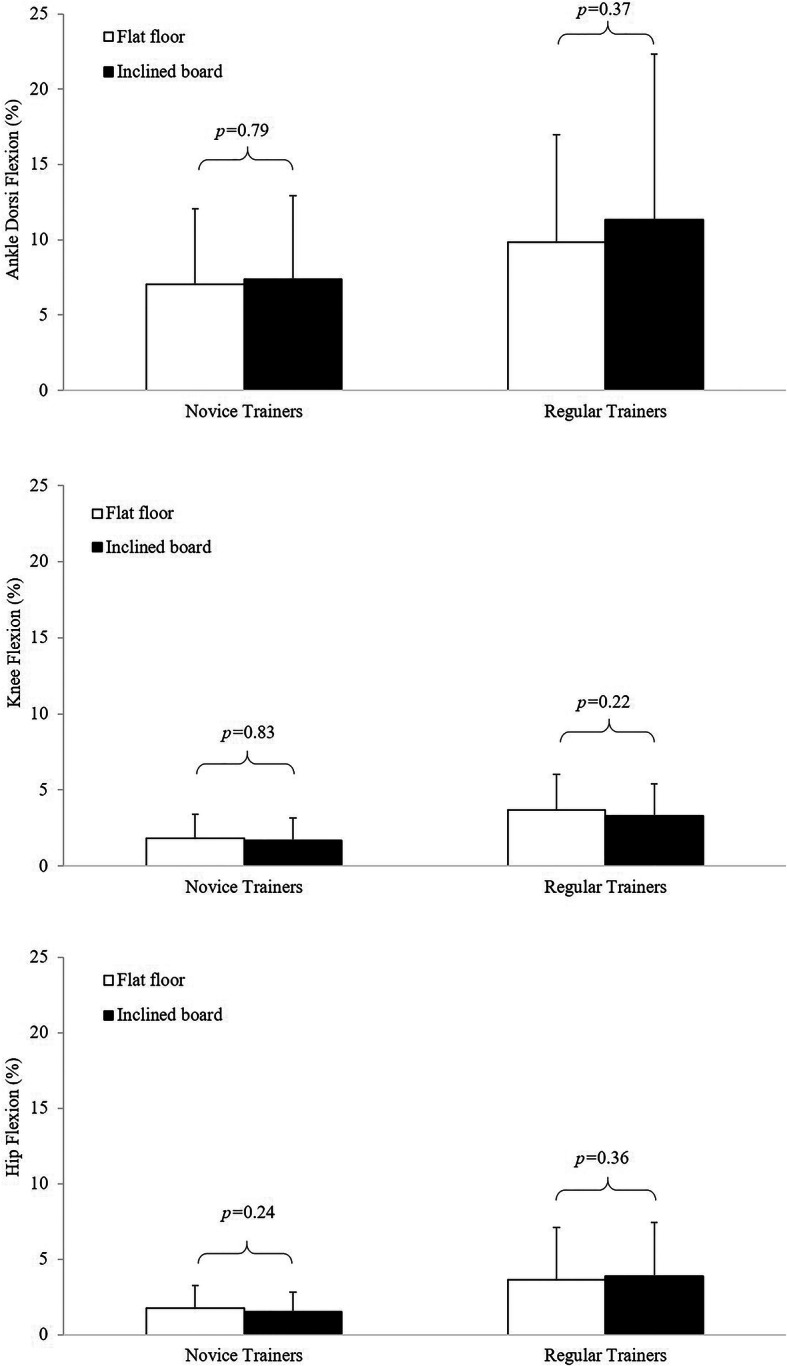
Ankle (top), knee (middle) and hip (bottom) joint symmetry index data based on maximum flexion angles for the novice and regular weight trainers

**Fig. 3:**
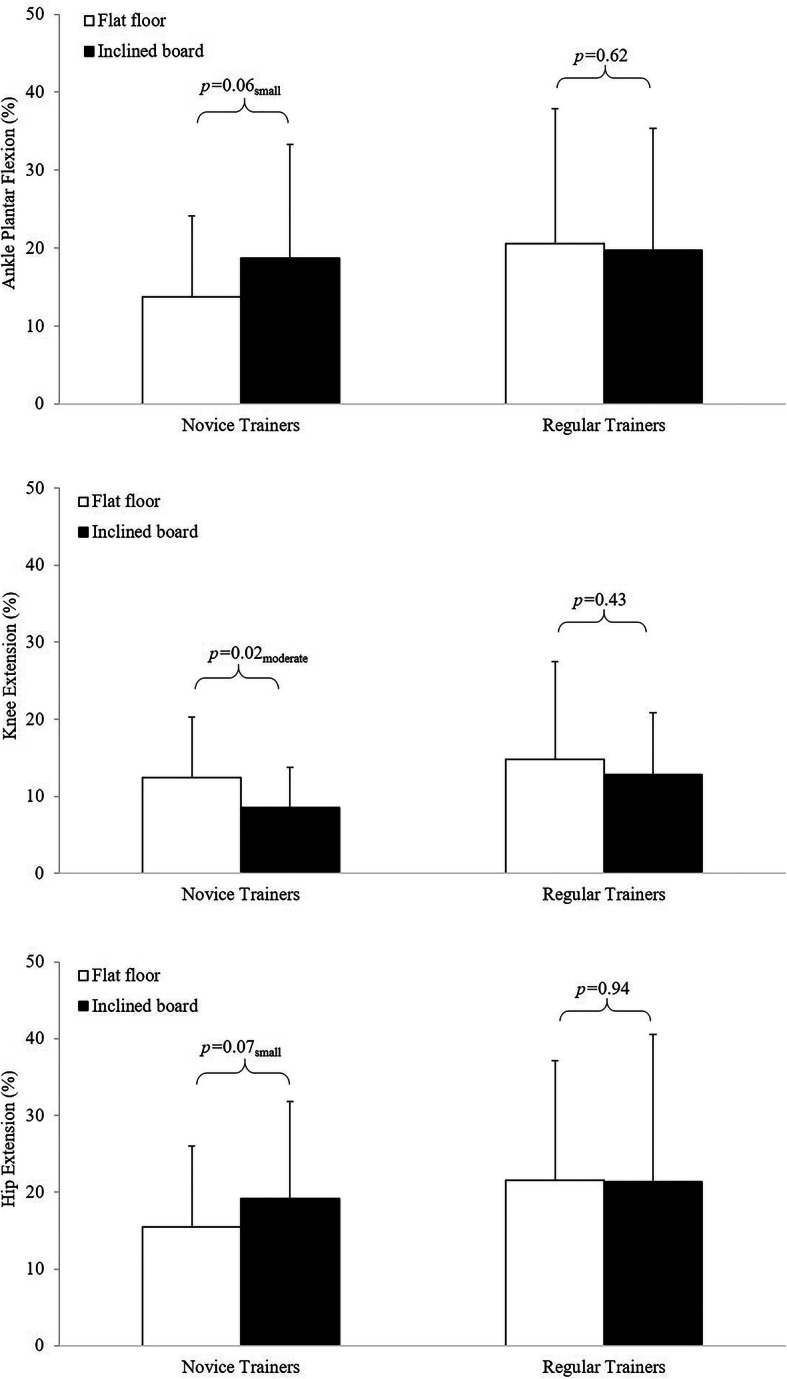
Ankle (top), knee (middle) and hip (bottom) joint symmetry index data based on maximum joint extension moments for the novice and regular weight trainers (*small* and *moderate* are descriptors of the relative magnitudes of the effect sizes for these

Analyses of discrete data indicated *trivial*, non-significant differences in peak joint flexion symmetry between *FL* and *EH* conditions (Fig. 2 and Table S1 in Supplementary materials). These discrete analyses also revealed greater bilateral asymmetry in peak ankle dorsi flexion than in peak knee and hip flexion. The regular weight training group also presented typically with greater bilateral asymmetry in these discrete angular displacement data than the novice trainers. Similar analyses of the peak joint moments showed *small* to *moderate* differences in bilateral symmetry between conditions, but only for the novice participants (Fig. 3 and Table S2 in Supplementary materials). Analyses of SI data indicated *trivial*, non-significant differences between *FL* and *EH* conditions for both the novice (*FL* = 3.4 ± 9.6%, *EH* = 4.4 ± 10.8%, *P* = 0.40. *g* = 0.09) and regular weight trainers (*FL* = 2.8 ± 10.9%, *EH* = 3.2 ± 10.1%, *P* = 0.79, *g* = 0.03).

**Fig. 4:**
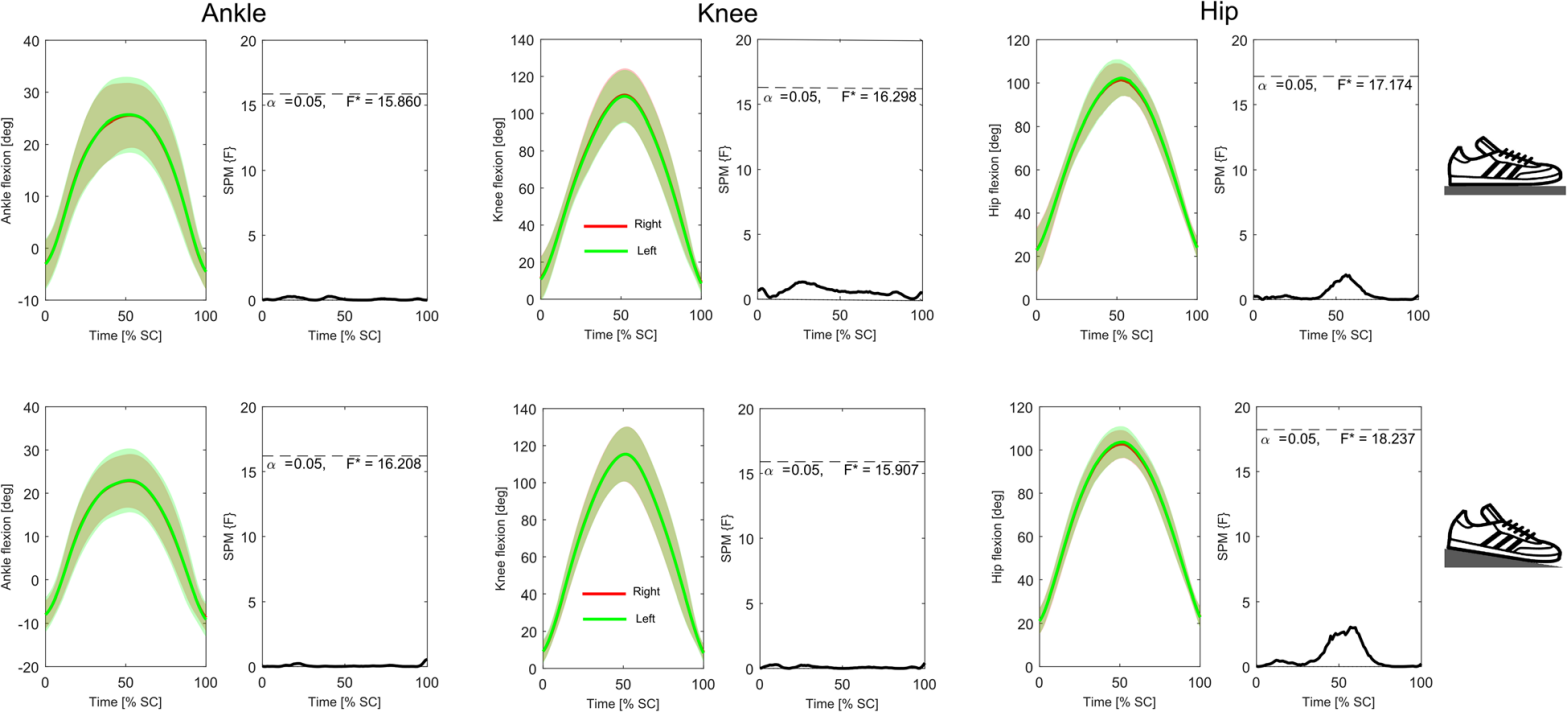
Time-series mean and standard deviation clouds representing the ankle (left), knee (middle) and hip (right) joint angles (°) for the novice weight trainers during both the flat floor (*FL*, top) and elevated heels (*EH*, bottom) squat conditions together with SPM data. Red lines represent data for the right leg, while the green lines are for the left leg

**Fig. 5:**
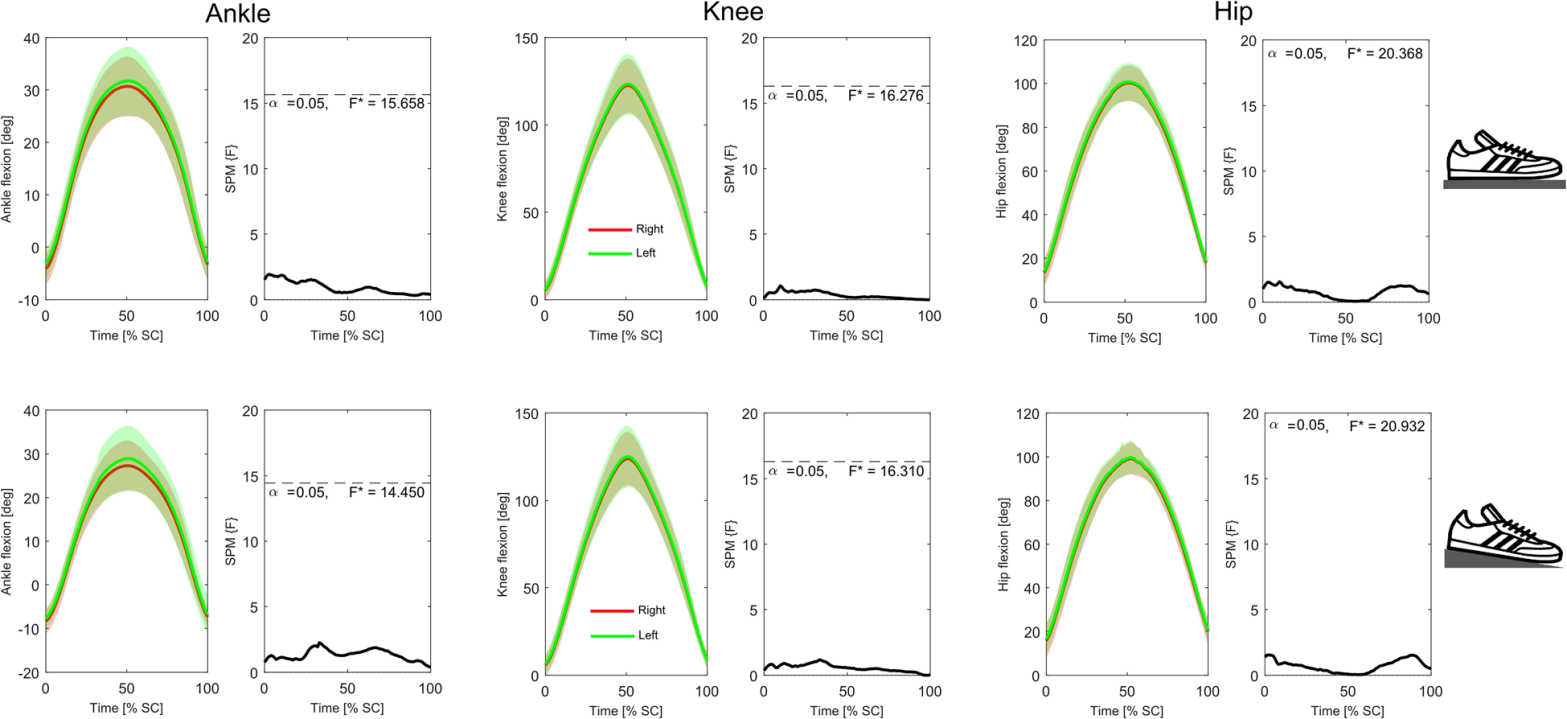
Time-series mean and standard deviation clouds representing the ankle (left), knee (middle) and hip (right) joint angles (°) for the regular weight trainers during both the flat floor (*FL*, top) and elevated heels (*EH*, bottom) squat conditions together with SPM data. Red lines represent data for the right leg, while the green lines are for the left leg

**Fig. 6:**
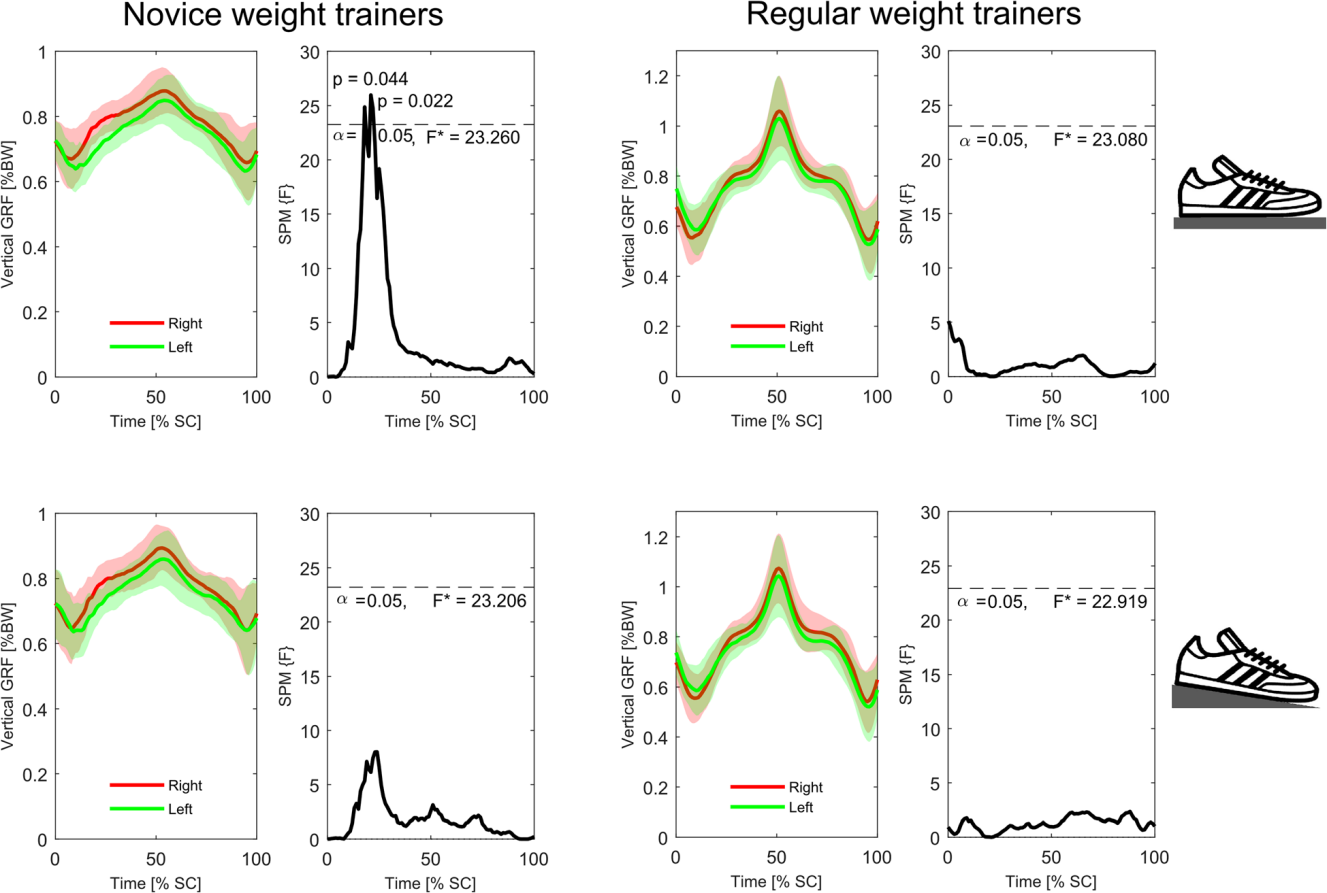
Time-series mean and standard deviation clouds representing the vertical ground reaction forces (%BW) for the novice (left) and regular weight trainers (right) during both the flat floor (*FL*, top) and elevated heels (*EH*, bottom) squat conditions together with SPM data. Red lines represent data for the right leg, while the green lines are for the left leg

**Fig. 7:**
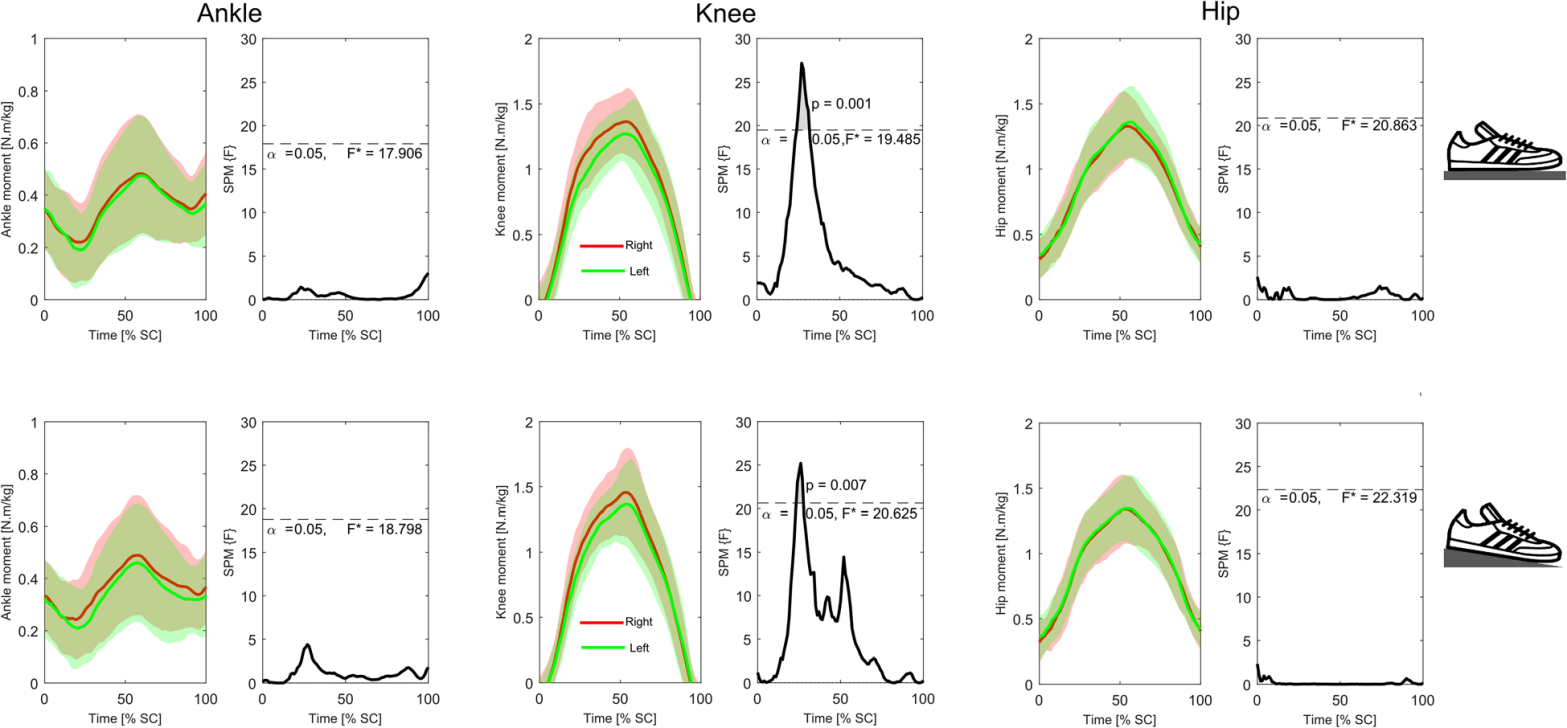
Time-series mean and standard deviation clouds representing the ankle (left), knee (middle) and hip (right) joint moments (Nm/kg) for the novice weight trainers during both the flat floor (*FL*, top) and elevated heels (*EH*, bottom) squat conditions together with SPM data. Red lines represent data for the right leg, while the green lines are for the left leg

**Fig. 8:**
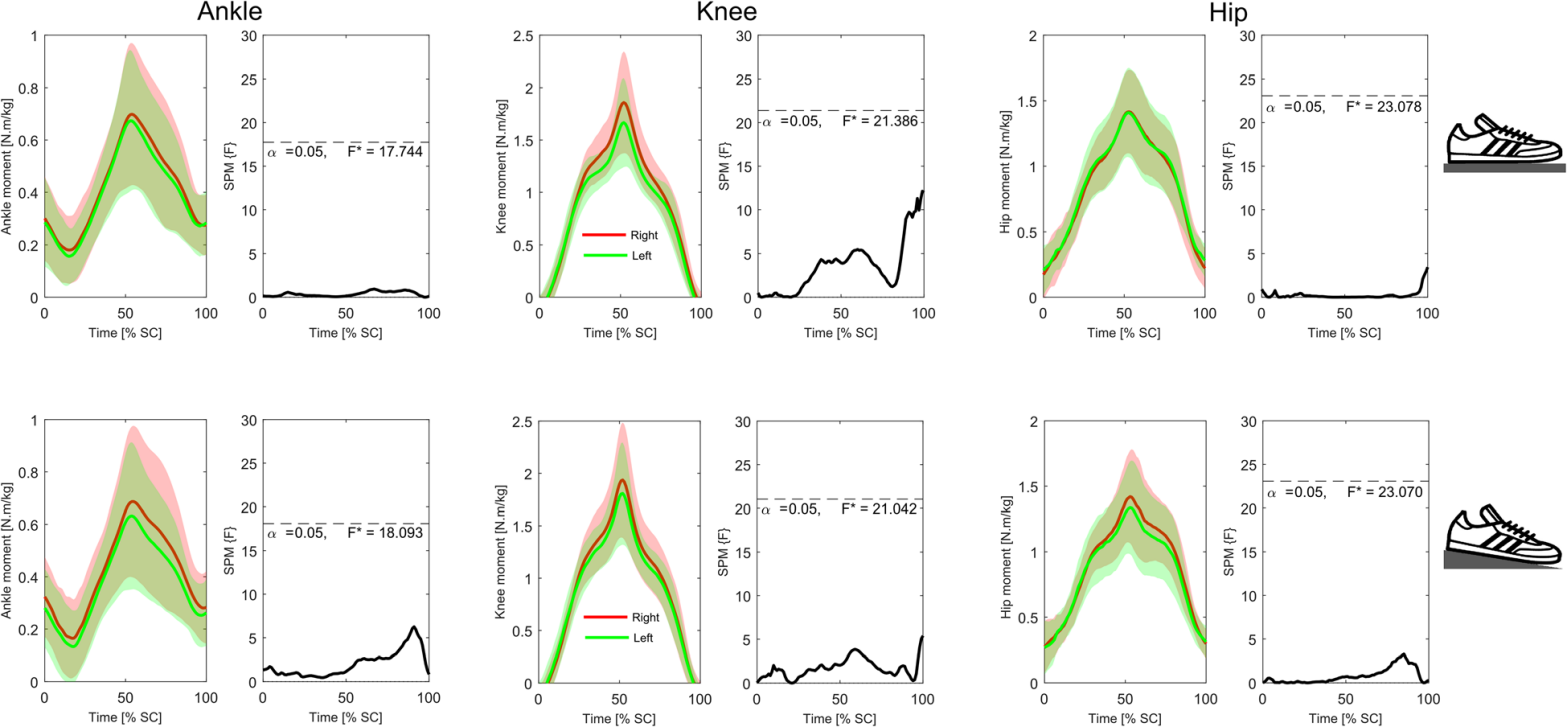
Time-series mean and standard deviation clouds representing the ankle (left), knee (middle) and hip (right) joint moments (Nm/kg) for the regular weight trainers during both the flat floor (*FL*, top) and elevated heels (*EH*, bottom) squat conditions together with SPM data. Red lines represent data for the right leg, while the green lines are for the left leg

Analysis of the time-series kinematic data (Figs. 4 and 5) showed that neither group presented with significant differences in bilateral symmetry in joint displacement at any stage of the squat cycle during either the *FL* or *EH* conditions. Conversely, similar analyses of GRF_vert_ data (Fig. 6) indicated that in the *FL* condition the novice weight trainers presented with significant bilateral asymmetries during the eccentric phase of the squat (approximately 25–35% through the cycle), with these differences becoming non-significant during the *EH* condition. These differences were also present in the novice weight trainer’s knee extension joint moments during both conditions (Fig. 7), although the duration of asymmetry decreased during the *EH* squats. In contrast to the novice group, the regular weight trainers did not display significant bilateral symmetries in their joint kinetics or GRF_vert_ time-series data in either condition (Figs. 4 and 8).

Time series representing frontal plane moments around the right and left joints of the lower limb for the two studied group as well as for each foot position (FL and EH) are presented in Figs. S1 and S2 (Supplementary materials).

## Discussion

This investigation examined the influence of elevating the heels on lower limb symmetry during moderate-load high-bar squatting in both novice and regular weight trainers. Analyses were based on quantifying symmetry using a combination of traditional discrete (e.g. bilateral differences in maximal joint displacement) and time-series data (e.g. joint moments throughout the squat cycle). Our key findings indicate that performing the high-bar back squat with *EH* alters the degree of symmetry in GRF_vert_ and joint moment data, particularly during the eccentric phase of the movement in novice but not regular weight trainers.

The *trivial* non-significant bilateral asymmetries in SI data are consistent with previous research reporting that bilateral differences in GRF_vert_ are common in asymptomatic individuals during both loaded and unloaded squats [7, 35, 36, 39]. Although our regular weight trainers were more symmetrical in these data than the novices, the overall levels of asymmetry were between 3 and 4% only and so are unlikely to be functionally meaningful [11, 12, 14]. It would also appear that SI data are largely unaffected by elevating the heels, with any potential individual asymmetry in ankle mobility not translating into peak right/left weight distribution during the squats. In stark contrast to these discrete SI data, time-series analyses of GRF_vert_ highlight significant asymmetries within the novice weight trainers during the *FL* condition. These asymmetries were typically in the order of 0.1 BW (about 15%) and occurred approximately mid-way though the eccentric phase of the squat. Not only are these values well in excess of those from discrete SI data, but asymmetries of this magnitude are at the threshold used to define injury risk [12, 14].

Discrete lower limb symmetry data for maximum knee and hip flexion angles were within 3% regardless of the heel lift condition. Although ankle dorsi flexion symmetry data were three to four times greater than at both the knee and hip joints, these data were still lower than the 15% injury risk threshold [11, 12, 14]. These differences in joint flexion symmetry are consistent with research on sit-to-stand tasks in healthy participants showing that asymmetries are typically greater in the ankles than at the knees and/or hips during these tasks [56]. Lower limb joint flexion symmetry also appeared to be largely unaffected by the heel lift condition throughout the entire squat cycle in both groups. Accordingly, while studies report that elevating the heels alter lower limb kinematics [27, 44–46, 57] any potential benefits from the *EH* condition do not appear to translate into changes in joint flexion symmetry.

A key finding in this investigation was the proportionally high levels of asymmetry in discrete lower limb joint extension moment data, with several values exceeding the 15% injury risk threshold [11, 12, 14]. The influence of the *EH* condition on these discrete data was more pronounced in the novice weight training group, although the overall levels of asymmetry were typically greater in the regular weight trainers than in the novices. The absence of an effect of *EH* on bilateral symmetry coupled with the greater squat depths achieved by the regular weight trainers suggests that symmetry in any of the measures used in this investigation occurs independently of ankle mobility in this group. The asymmetries in time-series knee extension moments demonstrated by the novice weight trainers during the eccentric phase of the *FL* squats also appear to be independent of ankle mobility as the *EH* condition had minimal effect on these data. These knee extension asymmetries therefore seem to be controlled primarily by asymmetries in GRF_vert_, as joint flexion data were highly symmetrical. The loads of just 50% BM used in our study were relatively light for experienced weight trainers, so our results may not be representative of symmetry data, or the influence of raising the heels on symmetry data for back squats with heavier loads. In this respect, additional research is still required in this area to not only elucidate the role of loading and fatigue on lifting symmetry and the subsequent training responses, but also its potential role on injury mechanisms and frequency.

To our knowledge no other studies have used continuous analytical procedures to report on the timing of bilateral lower limb symmetry during the back squat exercise. However, the timing of these asymmetries during the squat eccentric phase is consistent with research reporting kinetic impulse asymmetry index data during the eccentric phase of a counter movement jump as being double that of the concentric phase [58]. The neuromuscular control required during eccentric contractions is complex, as the motor cortex must increase excitability to compensate for increases in inhibitory neural drive from spinal reflex loops [59, 60]. These increases in cortical excitability during the eccentric phases of movements have been used by researchers to explain the improved neuromuscular control demonstrated by participants following periods of eccentric training [59, 61]. The absence of asymmetries during this phase in our regular weight training group suggests that bilateral lower limb symmetries might be representative of superior neuromuscular control in this group, although the cross-sectional nature of this investigation means that care should be taken to avoid over-generalising these findings.

Differences in the results from our discrete and time-series analyses highlight a key issue associated with relying solely on discrete data analysis techniques to assess bilateral symmetry. At the time of submission of this manuscript, the use of SPM (1D) techniques to assess time-series symmetry data had not been reported in the scientific literature. Although used typically to assess differences between conditions [62], these techniques also appear ideally suited for monitoring bilateral symmetry, with the capacity to assess symmetry over the entire time-series offering clear advantages over methods that rely on discrete data only.

The presented findings are limited to the young populations investigated in this study and so care should be taken to avoid generalising these data to older and/or more athletic groups. Similarly, the inexperience of our novice participants at performing high-bar back squats meant that we chose to load the bar at just 50% BM (i.e. not a function of RM), which will mean that the relative loads in our investigation differed between individuals. We also chose to limit analyses to assessing sagittal (i.e. flexion/extension) and vertical ground reaction force data and so additional care should be taken before generalising these findings to other non-sagittal movements. Although typical for research in this domain, the relatively small sample sizes in this study mean that there is the potential for type II errors in the results.

## Conclusions

This novel investigation adds to the body of knowledge in this field by using contemporary analytical procedures to assess the influence of elevating the heels on lower limb symmetry during the high bar squat. The key findings highlight that although a degree of bilateral lower limb asymmetry is common in individuals performing back squats, the degree of this symmetry is largely unaffected by raising the heels. Our results also show that when using traditional discrete measures of bilateral symmetry, the regular weight training group presented with more asymmetry than the novices, while analyses of time-series data indicated asymmetries in the novice group only. This project also emphasizes the limitations of traditional measures of bilateral symmetry and highlights advantages of quantifying symmetry in time-series data using SPM1D.

## Supplementary information

**Additional file 1: Table S1**, **Table S2**, and **Table S3.** Mean and standard deviation (in brackets) of the maximum joint angles in the sagittal plane and the corresponding symmetry indices (SIs) for the lower limb joints in expert (regular) and novice weight lifters. FL: flat heels and EH: elevated heels. **Figure S1 and Figure S2.** Time-series mean and standard deviation clouds representing the ankle (left), knee (middle) and hip (right) joint moments (Nm/kg) in the frontal plane for the novice weight trainers during both the flat floor (*FL*, top) and elevated heels (*EH*, bottom) squat conditions together with SPM data. Red lines represent data for the right leg, while the green lines are for the left leg.

## Data Availability

The datasets used and/or analysed during the current study are available from the corresponding author on reasonable request.

## Abbreviations

SI: Symmetry Index
SPM: Statistical Parametric Mapping
ACL: Anterior Cruciate Ligament
ROM: Range of Motion
EH: Elevated Heels
FL: Flat Floor
RM: Single Repetition Maximum
BW: Body Weight
GRF: Ground Reaction Force
*g*: *Hedge’s g*
