# Supplementary material for: The effect of increasing heel height on lower limb symmetry during the back squat in trained and novice lifters
Source: BMC Sports Sci Med Rehabil. 2020 Jul 25;12:42. doi: 10.1186/s13102-020-00191-y (PMC7382835; doi:10.1186/s13102-020-00191-y)
Supplement: Supplementary file 1 — Additional file 1: Table S1, Table S2, and Table S3. Mean and standard deviation (in brackets) of the maximum joint angles in the sagittal plane and the corresponding symmetry indices (SIs) for the lower limb joints in expert (regular) and novice weight lifters. FL: flat heels and EH: elevated heels. Figure S1 and Figure S2. Time-series mean and standard deviation clouds representing the ankle (left), knee (middle) and hip (right) joint moments (Nm/kg) in the frontal plane for the novice weight trainers during both the flat floor (FL, top) and elevated heels (EH, bottom) squat conditions together with SPM data. Red lines represent data for the right leg, while the green lines are for the left leg. [file 13102_2020_191_MOESM1_ESM.docx]

**Supplementary materials**

**Table S1.** Mean and standard deviation (in brackets) of the maximum joint angles in the sagittal plane and the corresponding symmetry indices (SIs) for the lower limb joints in expert (regular) and novice weight lifters. FL: flat heels and EH: elevated heels.

| Joint | Group | Condition | Max Right Joint Angle(°) | Max Left Joint Angle(°) | SI Joint Angle (%) |
| --- | --- | --- | --- | --- | --- |
| Ankle | Novice | FL | 26.10 (5.92) | 26.34 (6.94) | 7.04 (5.02) |
|  | Novice | EH | 23.22 (6.37) | 23.37 (7.46) | 7.40 (5.54) |
|  | Expert | FL | 30.89 (5.73) | 31.93 (6.58) | 9.86 (7.10) |
|  | Expert | EH | 27.51 (5.67) | 29.17 (7.47) | 11.32 (11.02) |
| Knee | Novice | FL | 111.77 (13.11) | 111.16 (12.73) | 1.80 (1.60) |
|  | Novice | EH | 116.61 (15.58) | 116.61 (15.52) | 1.70 (1.46) |
|  | Expert | FL | 123.05 (15.69) | 123.81 (17.49) | 3.66 (2.36) |
|  | Expert | EH | 124.56 (15.39) | 125.76 (17.55) | 3.28 (2.12) |
| Hip | Novice | FL | 102.49 (6.25) | 103.4 (7.05) | 1.78 (1.48) |
|  | Novice | EH | 103.37 (6.46) | 104.27 (7.27) | 1.52 (1.30) |
|  | Expert | FL | 100.73 (8.17) | 101.34 (8.65) | 3.64 (3.48) |
|  | Expert | EH | 100.16 (7.91) | 100.72 (8.32) | 3.88 (3.56) |

**Table S2.** Mean and standard deviation (in brackets) of the maximum joint moments in the sagittal plane and the corresponding symmetry indices (SIs) for the lower limb joints in expert (regular) and novice weight lifters. FL: flat heels and EH: elevated heels.

| Joint | Group | Condition | Max Right Joint Moment (N.m/kg) | Max Left Joint Moment (N.m/kg) | SI Joint Moment (%) |
| --- | --- | --- | --- | --- | --- |
| Ankle | Novice | FL | 0.09 (0.04) | 0.10 (0.04) | 32.33 (22.87) |
|  | Novice | EH | 0.11 (0.05) | 0.11 (0.07) | 35.70 (30.41) |
|  | Expert | FL | 0.10 (0.05) | 0.12 (0.08) | 50.13 (38.65) |
|  | Expert | EH | 0.11 (0.05) | 0.12 (0.05) | 49.76 (35.24) |
| Knee | Novice | FL | 0.31 (0.12) | 0.37 (0.12) | 34.46 (29.34) |
|  | Novice | EH | 0.31 (0.09) | 0.39 (0.14) | 34.04 (25.04) |
|  | Expert | FL | 0.49 (0.15) | 0.53 (0.19) | 21.69 (14.30) |
|  | Expert | EH | 0.52 (0.17) | 0.53 (0.21) | 21.80 (16.82) |
| Hip | Novice | FL | 0.29 (0.12) | 0.32 (0.11) | 19.85 (18.09) |
|  | Novice | EH | 0.30 (0.12) | 0.33 (0.11) | 23.22 (20.85) |
|  | Expert | FL | 0.29 (0.07) | 0.36 (0.10) | 30.98 (20.59) |
|  | Expert | EH | 0.32 (0.08) | 0.38 (0.11) | 30.01 (18.81) |

**Table S3.** Mean and standard deviation (in brackets) of the maximum vertical ground reaction force (GRF) and the corresponding symmetry indices (SIs) for expert (regular) and novice weight lifters. FL: flat heels and EH: elevated heels.

| Group | Condition | Max Right Foot GRF (%BW) | Max Left Foot GRF (%BW) | SI GRF (%) |
| --- | --- | --- | --- | --- |
| Novice | FL | 0.88 (0.07) | 0.85 (0.07) | 3.38 (9.50) |
| Novice | EH | 0.89 (0.07) | 0.86 (0.09) | 4.37 (10.75) |
| Expert | FL | 1.06 (0.14) | 1.03 (0.17) | 2.83 (10.88) |
| Expert | EH | 1.07 (0.14) | 1.04 (0.16) | 3.20 (10.08) |


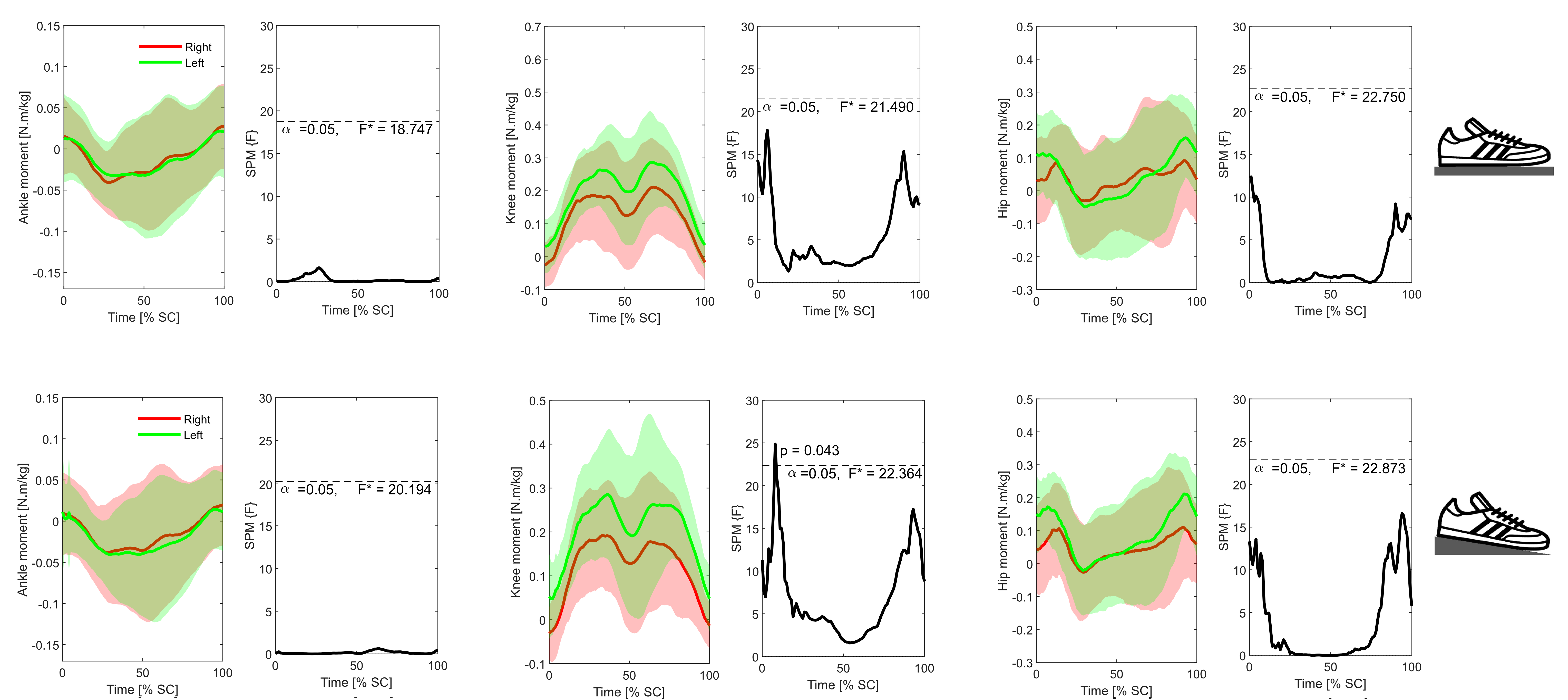


**Figure S1.** Time-series mean and standard deviation clouds representing the ankle (left), knee (middle) and hip (right) joint moments (Nm/kg) in the frontal plane for the novice weight trainers during both the flat floor (*FL*, top) and elevated heels (*EH*, bottom) squat conditions together with SPM data. Red lines represent data for the right leg, while the green lines are for the left leg.


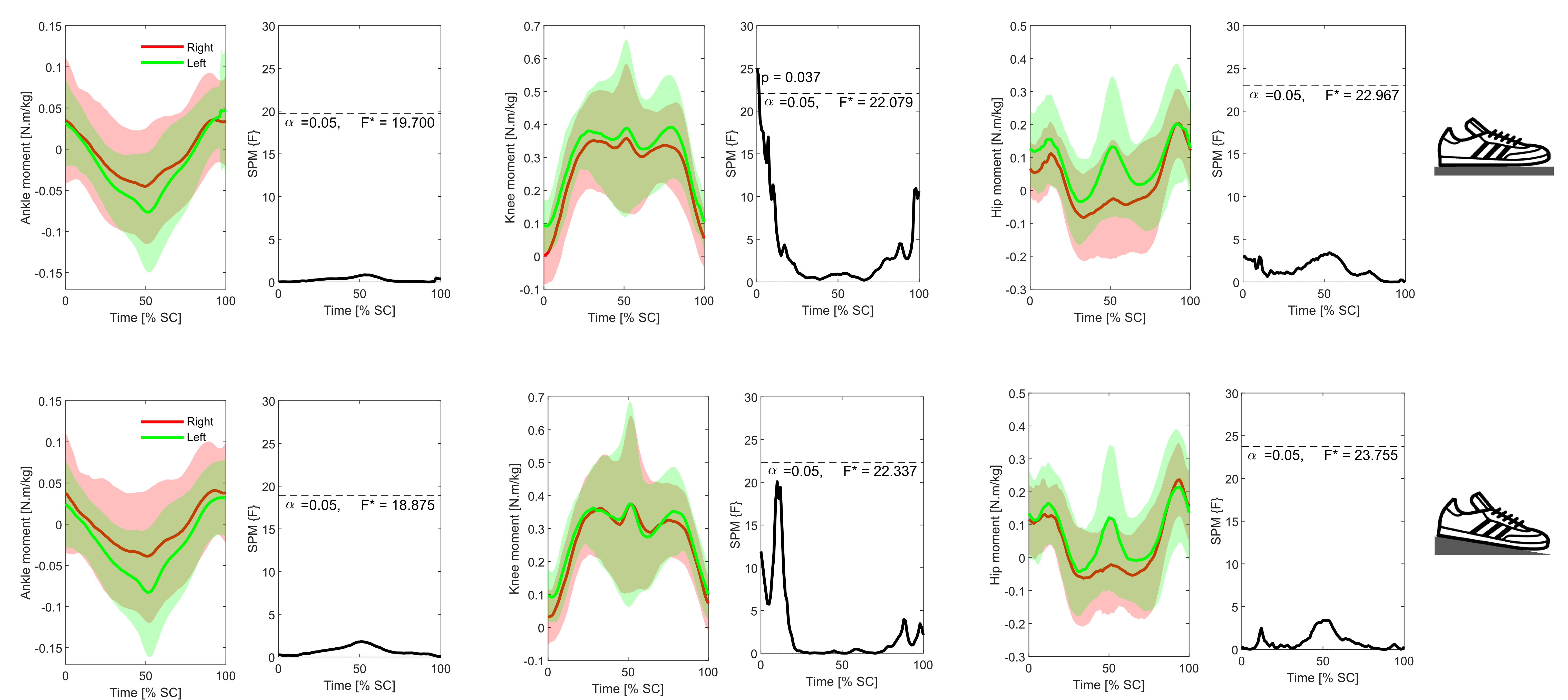


**Figure S2.** Time-series mean and standard deviation clouds representing the ankle (left), knee (middle) and hip (right) joint moments (Nm/kg) in the frontal plane for the regular weight trainers during both the flat floor (*FL*, top) and elevated heels (*EH*, bottom) squat conditions together with SPM data. Red lines represent data for the right leg, while the green lines are for the left leg.
